# Supplementary material for: Improving polygenic prediction from summary data by learning patterns of effect sharing across multiple phenotypes
Source: PLoS Genet. 2025 Jan 7;21(1):e1011519. doi: 10.1371/journal.pgen.1011519 (PMC11741642; doi:10.1371/journal.pgen.1011519)
Supplement: S1 Table — For LDpred2-auto, the statistics are based on the sum of runtime across phenotypes. Each method was run using 4 CPUs. (PDF) [file pgen.1011519.s001.pdf]

Supplementary Table 1: Summary statistics for runtime (in seconds) on chromosome 10 for the "Shared Effects in Subgroups" scenario. For *LDpred2-auto*, the statistics are based on the sum of runtime across phenotypes. Each method was run using 4 CPUs.

	**Methods**		
	LDpred2-auto	*SmvBayesC*	*mr.mash-rss*
Minimum	235.4	2677.5	1409.1
1st quartile	279.0	2985.2	1627.2
Median	287.5	3006.3	1818.7
Mean	285.6	3054.4	1816.6
3rd quartile	296.5	3223.0	1929.8
Maximum	317.8	3371.6	2309.4
