# Supplementary material for: Improving polygenic prediction from summary data by learning patterns of effect sharing across multiple phenotypes
Source: PLoS Genet. 2025 Jan 7;21(1):e1011519. doi: 10.1371/journal.pgen.1011519 (PMC11741642; doi:10.1371/journal.pgen.1011519)
Supplement: S3 Table — (PDF) [file pgen.1011519.s003.pdf]

Supplementary Table 3: Mean h_g^2 across training sets for the 16 blood cell traits in the full UK Biobank data.

**Phenotype**	$\mathbf{h_g^2}$
Red Blood Cell Counts (RBC#)	0.23
Haemoglobin Concentration (HGB)	0.19
Mean Corpuscular Volume (MCV)	0.28
Red Blood Cell Volume Distribution Width (RDW)	0.22
Mean Sphered Cell Volume (MSCV)	0.23
Reticulocyte Percentage (RET%)	0.21
High Light Scatter Reticulocytes Percentage (HLR%)	0.22
Platelet Count (PLT#)	0.31
Plateletcrit (PCT)	0.26
Platelet Distribution Width (PDW)	0.24
White Blood Cell Count (WBC#)	0.20
Lymphocyte Percentage (LYMPH%)	0.16
Monocyte Percentage (MONO%)	0.20
Neutrophil Percentage (NEUT%)	0.16
Eosinophil Percentage (EO%)	0.20
Basophil Percentage (BASO%)	0.05
