# Supplementary material for: Improving polygenic prediction from summary data by learning patterns of effect sharing across multiple phenotypes
Source: PLoS Genet. 2025 Jan 7;21(1):e1011519. doi: 10.1371/journal.pgen.1011519 (PMC11741642; doi:10.1371/journal.pgen.1011519)
Supplement: S1 Text — Detailed description of the methods, including: derivations of the mr.mash-rss algorithms; data preparation; simulations; methods compared; data analysis. (PDF) [file pgen.1011519.s007.pdf]

Improving polygenic prediction from summary data by learning patterns of effect sharing across multiple phenotypes—S1 Text

Deborah Kunkel[1*], Peter Sørensen [2], Vijay Shankar[3], Fabio Morgante[3,4*]

1 *School of Mathematical and Statistical Sciences, Clemson University, Clemson, South Carolina, United States of America*
2 *Center for Quantitative Genetics and Genomics, Aarhus University, Aarhus, Denmark*
3 *Center for Human Genetics, Clemson University, Greenwood, South Carolina, United States of America*
4 *Department of Genetics and Biochemistry, Clemson University, Clemson, South Carolina, United States of America*

Conventions used in mathematical expressions

For the mathematical expressions below, we denote matrices as bold, uppercase letters (e.g., $\mathbf{A}$), column vectors as bold, lowercase letters ($\mathbf{a}$), and scalars are written in plain, lowercase letters (a). We use $\mathbb{R}^{m \times n}$ to denote the set of real $m \times n$ matrices, we use $\mathbf{I}_n$ to denote the $n \times n$ identity matrix, $\mathbf{1}_n$ is a column-vector of ones of length n, and $\text{tr}(\mathbf{A}) = \sum_{i=1}^{n} a_{ii}$ denotes the trace of $n \times n$ matrix $\mathbf{A}$.

For a matrix $\mathbf{A}$, let $\mathbf{A}_{\{i,j\}}$ denote its i, j^{th} element, let $\mathbf{A}_{\{,j\}}$ and $\mathbf{A}_{\{j,\}}$ denote the jth column and j^{th} row of $\mathbf{A}$, respectively. Further, let $\mathbf{A}_{\{,-j\}}$ denote the matrix with its jth column removed and let $\mathbf{A}_{\{-j,\}}$ denote the matrix with its jth row removed. For a matrix of the form $\mathbf{A}^\intercal\mathbf{B}$ with $\mathbf{A}$ having j^{th} column $\mathbf{a}_j$, the quantity $\mathbf{a}_j^\intercal\mathbf{B}$ can be obtained by subsetting as: $\mathbf{a}_j^\intercal\mathbf{B} = \mathbf{A}^\intercal\mathbf{B}_{\{j,\}}$ and $\mathbf{a}_j^\intercal\mathbf{a}_j = \mathbf{A}^\intercal\mathbf{A}_{\{j,j\}}$.

For describing Bayesian calculations, our convention is to use the "0" subscript to indicate priors and the "1" subscript to indicate posteriors.

1. *mr.mash* model

In *mr.mash* and *mr.mash-rss*, the response follows a Bayesian multivariate multiple regression model; that is,

$$\begin{aligned} \boldsymbol{Y} &= \boldsymbol{X}\boldsymbol{B} + \boldsymbol{E} \\ \boldsymbol{E} &\sim MN_{n\times r}\left(\mathbf{0}, \boldsymbol{I}_n, \boldsymbol{V}\right), \end{aligned} \tag{5}$$

where $\boldsymbol{Y}$ is an $n \times r$ matrix, $\boldsymbol{X}$ is an $n \times p$ matrix, $\boldsymbol{B}$ is a $p \times r$ matrix, and $\boldsymbol{V}$ is an $r \times r$ matrix. The effects of the jth variable is denoted by the r-vector $\boldsymbol{b}_j$, where $\boldsymbol{b}_j^\intercal$ is the jth row of $\boldsymbol{B}$. The prior on $\boldsymbol{B}$ is

$$\boldsymbol{b}_j \mid \boldsymbol{w}_0, \mathscr{S}_0 \sim \sum_{k=1}^{K} w_{0,k} N_r(\mathbf{0}, \boldsymbol{S}_{0,k}), \quad j = 1, \ldots, p. \tag{6}$$

2. Fitting the model with sufficient statistics

In the next section we describe methods for fitting the *mr.mash* model that rely on the data only through $\boldsymbol{X}^\intercal\boldsymbol{X}$, $\boldsymbol{X}^\intercal\boldsymbol{Y}$, and $\boldsymbol{Y}^\intercal\boldsymbol{Y}$, the sufficient statistics for the model in (5).

2.1. Update of $\mathbf{B}$.

We approximate the posterior distribution of $\boldsymbol{B}$ through variational inference, a method that approximates the posterior through optimization rather than sampling, as in Monte Carlo methods. A class of distributions is specified for the approximate posterior, denoted by $q(\boldsymbol{B})$. The optimal q from within this class is found by minimizing the Kullback-Leibler divergence (D_{KL}) of the approximation from the true posterior or, equivalently, by maximizing the evidence lower bound (ELBO) with respect to q. The ELBO is defined to be

$$F(q; \boldsymbol{V}, \boldsymbol{w}_0) := \log(p(\boldsymbol{Y}|\boldsymbol{X}, \boldsymbol{V})) - D_{KL}\left(q(\boldsymbol{B}) \mid\mid p(\boldsymbol{B}|\boldsymbol{X}, \boldsymbol{Y}, \boldsymbol{V}, \boldsymbol{w}_0)\right) \tag{7}$$

where the first term is the log marginal likelihood of the model and the second term is the Kullback-Leibler divergence of the approximate posterior from the true posterior.

Following [11], in *mr.mash-rss*, we assume a mean-field approximation for $q(\boldsymbol{B})$; that is, we restrict the approximate posterior to take the form:

$$q(\boldsymbol{B}) = \prod_{j=1}^{p} q_j(\boldsymbol{b}_j), \tag{8}$$

where the q_j is a probability distribution on the jth row of $\boldsymbol{B}$. Subject to this constraint, the ELBO can be expressed in a more tractable form:

$$F(q_1, \ldots, q_p; \boldsymbol{V}, \boldsymbol{w}_0) = E_q \left[\log(p(\boldsymbol{Y}|\boldsymbol{X}, \boldsymbol{V}, \boldsymbol{B}))\right] + \sum_{j=1}^{p} D_{KL}\left(q_j(\boldsymbol{b}_j)||g(\boldsymbol{b}_j)\right) \tag{9}$$

where the expectation in the first term is with respect to the approximate posterior, q, and $g(\boldsymbol{b}_j)$ denotes the prior on $\boldsymbol{b}_j$.

We maximize $F(q; \boldsymbol{V}, \boldsymbol{w}_0)$ sequentially with respect to each q_j, holding $q_{j'}$ constant for $j' \neq j$. It can be shown that the optimal q_j is a K-component mixture-of-Normals of the form:

$$q_j(\boldsymbol{b}_j) = \sum_{k=1}^{K} w_{1,k}^{(j)} \mathrm{N}_r(\boldsymbol{b}_{1,k}^{(j)}, \mathbf{S}_{1,k}^{(j)}). \tag{10}$$

The parameters of the mixture in (10) are functions of the coefficients of a least-squares multivariate regression of $\bar{\boldsymbol{R}}_{(j)}$ on $\boldsymbol{x}_j$, where $\bar{\boldsymbol{R}}_{(j)}$ denotes the residuals with the effect of $\boldsymbol{b}_j$ removed; *i.e.*, $\bar{\boldsymbol{R}}_{(j)}$ is the $n \times r$ matrix whose i^{th} row is $\boldsymbol{Y}_{\{i,\}} - \sum_{j' \neq j} x_{ij'} \boldsymbol{b}_{j'}^{\intercal}$. The weight, mean, and covariance for the kth component and j^{th} predictor are given by the following expressions:

$$\begin{aligned} \boldsymbol{S}_{1,k}^{(j)} &= \left(\boldsymbol{S}_{0,k}^{-1} + \widehat{\boldsymbol{S}}_j^{-1}\right)^{-1} \\ \boldsymbol{b}_{1,k}^{(j)} &= \boldsymbol{S}_{1,k}^{(j)} \widehat{\boldsymbol{S}}_j^{-1} \widehat{\boldsymbol{b}}_j \\ w_{1,k}^{(j)} &\propto w_{0,k} \frac{|\widehat{\boldsymbol{S}}_j|^{1/2}}{|\boldsymbol{S}_{0,k} + \widehat{\boldsymbol{S}}_j|^{1/2}} \exp\left(\frac{1}{2}\left(\boldsymbol{b}_{1,k}^{(j)}\right)^{\intercal}\left(\boldsymbol{S}_{1,k}^{(j)}\right)^{-1} \boldsymbol{b}_{1,k}^{(j)}\right), \end{aligned} \tag{11}$$

where $\widehat{\boldsymbol{S}}_j = \boldsymbol{V}/\boldsymbol{x}_j^{\intercal}\boldsymbol{x}_j$ and $\widehat{\boldsymbol{b}}_j^{\intercal} = \boldsymbol{x}_j^{\intercal}\bar{\boldsymbol{R}}_{(j)}/\boldsymbol{x}_j^{\intercal}\boldsymbol{x}_j$. The quantity $\boldsymbol{x}_j^{\intercal}\bar{\boldsymbol{R}}_{(j)}$ is the jth row of $\boldsymbol{X}^{\intercal}\bar{\boldsymbol{R}}_{(j)}$, which is computed as

$$\boldsymbol{X}^{\intercal}\bar{\boldsymbol{R}}_{(j)} = \boldsymbol{X}^{\intercal}\boldsymbol{X}\bar{\boldsymbol{B}} - \boldsymbol{X}^{\intercal}\boldsymbol{Y} + \boldsymbol{X}^{\intercal}\boldsymbol{x}_j\bar{\boldsymbol{b}}_j^{\intercal}. \tag{12}$$

The quantity $\boldsymbol{X}^{\intercal}\boldsymbol{x}_j$ is the jth column of $\boldsymbol{X}^{\intercal}\boldsymbol{X}$ and can be computed by subsetting $\boldsymbol{X}^{\intercal}\boldsymbol{x}_j = \boldsymbol{X}^{\intercal}\boldsymbol{X}_{\{,j\}}$ and $\bar{\boldsymbol{B}}$ is the $p \times r$ matrix whose j^{th} row is $\bar{\boldsymbol{b}}_j^{\intercal}$, defined below in (14).

We define the function BMSR-mix-ss to be one that returns the coefficients $\boldsymbol{b}_{1,k}^{(j)}$, $w_{1,k}^{(j)}$, and $\boldsymbol{S}_{1,k}^{(j)}$, $k = 1, \ldots, K$ of the distribution q_j, given $\boldsymbol{x}_j^{\intercal}\boldsymbol{x}_j$, $\boldsymbol{x}_j^{\intercal}\bar{\boldsymbol{R}}_{(j)}$, and the prior:

$$\text{BMSR-mix-ss}(\boldsymbol{x}_j^{\intercal}\boldsymbol{x}_j, \boldsymbol{x}_j^{\intercal}\bar{\boldsymbol{R}}_{(j)}, \boldsymbol{w}_0, \mathscr{S}_0) := \left(w_{1,1}^{(j)} \ldots, w_{1,K}^{(j)}, \boldsymbol{b}_{1,1}^{(j)} \ldots, \boldsymbol{b}_{1,K}^{(j)}, \boldsymbol{S}_{1,1}^{(j)} \ldots, \boldsymbol{S}_{1,K}^{(j)}\right) \tag{13}$$

Note: in the *mr.mash-rss* algorithm, the updates use BMSR-mix-ss$(\boldsymbol{x}_j^{\intercal}\boldsymbol{x}_j, \boldsymbol{x}_j^{\intercal}\bar{\boldsymbol{R}}_{(j)}, \boldsymbol{w}_0, \mathscr{S}_0)$ to iteratively update $\boldsymbol{b}_j$ for $j = 1, \ldots, p$.

The parameters calculated in (13) are used to updated $\boldsymbol{b}_j$ by its posterior mean $\bar{\boldsymbol{b}}_j := E_{q_j}(\boldsymbol{b}_j)$. The update is given below as a function of the component means, covariances, and weights of the distribution in (10).

$$\begin{aligned} \bar{\boldsymbol{b}}_j &= \sum_{k=1}^{K} w_{1,k}^{(j)} \boldsymbol{b}_{1,k}^{(j)} \\ \boldsymbol{S}_j &= \sum_{k=1}^{K} w_{1,k}^{(j)} \left(\boldsymbol{S}_{1,k}^{(j)} + \boldsymbol{b}_{1,k}^{(j)}\left(\boldsymbol{b}_{1,k}^{(j)}\right)^{\intercal}\right) - \bar{\boldsymbol{b}}_j\bar{\boldsymbol{b}}_j^{\intercal} \end{aligned} \tag{14}$$

2.2. Update of $\boldsymbol{w}_0$.

The prior (6) is determined by the pre-specified covariance matrices, $\mathscr{S}_0$, and the prior weights given by $\boldsymbol{w}_0$. We assume that the elements of $\mathscr{S}_0$ are known, and we estimate $\boldsymbol{w}_0$ from the data by maximizing the ELBO. Its update, derived in [11], is

$$\widehat{w}_{0,k} = \frac{1}{p}\sum_{j=1}^{p} w_{1,k}^{(j)}, \quad k = 1, \ldots, K, \tag{15}$$

where the $w_{1,k}^{(j)}$, $j = 1, \ldots, p; k = 1, \ldots, K$ are obtained from BMSR-mix-ss defined in (13). We define the function UPDATE-WEIGHTS to be a function that outputs $\boldsymbol{w}_0$ according to the expressions (15) above.

$$\textsc{Update-Weights}(\boldsymbol{W}) := (\hat{w}_{0,1}, \ldots, \hat{w}_{0,K}), \tag{16}$$

2.3. Update of $\boldsymbol{V}$.

The update of $\boldsymbol{V}$ is also obtained by maximizing the ELBO with respect to $\boldsymbol{V}$. The update derived in [11] can be expressed in terms of sufficient statistics as follows:

$$\hat{\boldsymbol{V}} = \mathrm{ERSS}/n, \tag{17}$$

In the expression above, ERSS is equal the expected residual sum of squares, and it can be expressed using sufficient statistics as follows:

$$\begin{aligned} \mathrm{ERSS} &= E_q\left(||\boldsymbol{Y} - \boldsymbol{X}\boldsymbol{B}||^2\right) \\ &= \bar{\boldsymbol{R}}^\intercal \bar{\boldsymbol{R}} + \sum_{j=1}^{p} \boldsymbol{x}_j^\intercal \boldsymbol{x}_j \boldsymbol{S}_j \end{aligned} \tag{18}$$

where

$$\begin{aligned} \bar{\boldsymbol{R}}^\intercal \bar{\boldsymbol{R}} &:= \left(\boldsymbol{Y} - \boldsymbol{X}\bar{\boldsymbol{B}}\right)^\intercal \left(\boldsymbol{Y} - \boldsymbol{X}\bar{\boldsymbol{B}}\right) \\ &= \boldsymbol{Y}^\intercal\boldsymbol{Y} - \bar{\boldsymbol{B}}^\intercal\boldsymbol{X}^\intercal\boldsymbol{Y} - \boldsymbol{Y}^\intercal\boldsymbol{X}\bar{\boldsymbol{B}} + \bar{\boldsymbol{B}}^\intercal\boldsymbol{X}^\intercal\boldsymbol{X}\bar{\boldsymbol{B}}. \end{aligned} \tag{19}$$

We define the function UPDATE-RESID-COV-SS to be a function that outputs $\boldsymbol{V}$ according to the expressions (17) and (18) above.

$$\textsc{Update-Resid-Cov-ss}(\boldsymbol{X}^\intercal\boldsymbol{X}, \boldsymbol{X}^\intercal\boldsymbol{Y}, \boldsymbol{Y}^\intercal\boldsymbol{Y}, \bar{\boldsymbol{B}}, \boldsymbol{S}_1, \ldots, \boldsymbol{S}_p) := \hat{\boldsymbol{V}} \tag{20}$$

2.4. Calculation of the ELBO

From (9), the ELBO for the *mr.mash-rss* is

$$F(q_1, \ldots, q_p; \boldsymbol{V}, \boldsymbol{w}_0) = E_q\left[\log\left(p(\boldsymbol{Y} \mid \boldsymbol{X}, \boldsymbol{B}, \boldsymbol{V}, \boldsymbol{w}_0)\right] + \sum_{j=1}^{p} E_q\left[\log\left(\frac{p(\boldsymbol{b}_j)}{q_j(\boldsymbol{b}_j)}\right)\right], \tag{21}$$

with $p(\boldsymbol{b}_j)$ denoting the prior density of $\boldsymbol{b}_j$ and the second term representing the negative KL-divergence of p from q_j. The first term in (21) can be expressed with sufficient statistics as :

$$E_q\left[\log\left(p(\boldsymbol{Y} \mid \boldsymbol{X}, \boldsymbol{B}, \boldsymbol{V}, \boldsymbol{w}_0)\right] = c - \frac{n}{2}\log|V| - \frac{1}{2}\mathrm{tr}\left(\boldsymbol{V}^{-1}\bar{\boldsymbol{R}}^\intercal\bar{\boldsymbol{R}}\right) + \sum_{j=1}^{p}\mathrm{tr}\left(\boldsymbol{V}^{-1}\boldsymbol{S}_j\right)\boldsymbol{x}_j^\intercal\boldsymbol{x}_j \tag{22}$$

where the c represents terms that are constant with respect to q, $\boldsymbol{V}$, and $\boldsymbol{w}_0$, and $\bar{\boldsymbol{R}}^\intercal\bar{\boldsymbol{R}}$ is calculated using (19). The jth summand in the second term of (21) is

$$\begin{aligned} E_q\left[\log\left(\frac{q_j(\boldsymbol{b}_j)}{p(\boldsymbol{b}_j)}\right)\right] &= -D_{KL}(q_j(\boldsymbol{b}_j) \mid\mid p(\boldsymbol{b}_j)) \\ &= \log\left[\sum_{k=1}^{K} w_{0,k}\left(\frac{|\widehat{\boldsymbol{S}}_j|^{1/2}}{|\boldsymbol{S}_{0,k} + \widehat{\boldsymbol{S}}_j|^{1/2}}\right)\exp\left(-\frac{1}{2}\left(\boldsymbol{b}_{1,k}^{(j)}\right)^\intercal\left(\boldsymbol{S}_{1,k}^{(j)}\right)^{-1}\boldsymbol{b}_{1,k}^{(j)}\right)\right] + \\ &\quad \frac{1}{2}\left[\mathrm{tr}(\boldsymbol{V}^{-1}\left(-\bar{\boldsymbol{b}}_j\boldsymbol{x}_j^\intercal\bar{\boldsymbol{R}}_j - \bar{\boldsymbol{R}}_j^\intercal\boldsymbol{x}_j\bar{\boldsymbol{b}}_j^\intercal + (\boldsymbol{S}_j + \bar{\boldsymbol{b}}_j\bar{\boldsymbol{b}}_j^\intercal)\boldsymbol{x}_j^\intercal\boldsymbol{x}_j\right)\right] \end{aligned} \tag{23}$$

Algorithm 1 *mr.mash*-sufficient

Require: $\boldsymbol{X}^\intercal\boldsymbol{X}$, $\boldsymbol{X}^\intercal\boldsymbol{Y}$, and $\boldsymbol{Y}^\intercal\boldsymbol{Y}$.
Require: Set of K covariance matrices, $\mathscr{S}_0$.
Require: Initial estimates of the posterior mean coefficients, stored as a $p \times r$ matrix, $\bar{\boldsymbol{B}}$ whose j^{th} row is $\bar{\boldsymbol{b}}_j^\intercal$.
Require: Initial estimates of the prior mixture weights, $\boldsymbol{w}_0 = (w_{0,1}, \ldots, w_{0,K})$, and the $r \times r$ residual covariance matrix $\boldsymbol{V}$.
Require: Convergence threshold, tol ≥ 0, and an upper limit on the number of iterations, $t_{\max}$.

$t \leftarrow 0$
$\delta \leftarrow \infty$
$\text{ELBO}^{(0)} \leftarrow F(q; \boldsymbol{V}, \boldsymbol{w}_0)$
while $\delta >$ tol and $t < t_{\max}$ **do**
  $t \leftarrow t + 1$
  Compute $\boldsymbol{X}^\intercal\bar{\boldsymbol{R}} \leftarrow \boldsymbol{X}^\intercal\boldsymbol{Y} - \boldsymbol{X}^\intercal\boldsymbol{X}\bar{\boldsymbol{B}}$
  Initialize $\boldsymbol{W}$ to a $p \times K$ matrix of zeros
  for j in $1, \ldots, p$ **do**
    Remove variable j from "expected residuals", $\boldsymbol{X}^\intercal\bar{\boldsymbol{R}}_{(j)} \leftarrow \boldsymbol{X}^\intercal\bar{\boldsymbol{R}} + \boldsymbol{X}^\intercal\boldsymbol{x}_j\bar{\boldsymbol{b}}_j^\intercal$
    $\left(w_{1,1}^{(j)}, \ldots, w_{1,K}^{(j)}, \boldsymbol{b}_{1,1}^{(j)}, \ldots, \boldsymbol{b}_{1,K}^{(j)}, \boldsymbol{S}_{1,1}^{(j)}, \ldots, \boldsymbol{S}_{1,K}^{(j)}\right) \leftarrow \text{BMSR-mix-ss}(\boldsymbol{x}_j^\intercal\boldsymbol{x}_j, \boldsymbol{x}_j^\intercal\bar{\boldsymbol{R}}_{(j)}, \boldsymbol{V}, \mathscr{S}_0, \boldsymbol{w}_0)$ ▷ See (13).
    Compute posterior mean, $\bar{\boldsymbol{b}}_j = \sum_{k=1}^K w_{1,k}^{(j)}\boldsymbol{b}_{1,k}^{(j)}$
    Compute posterior covariance, $\boldsymbol{S}_j = \sum_{k=1}^K w_{1,k}^{(j)}\left[\boldsymbol{b}_{1,k}^{(j)}\left(\boldsymbol{b}_{1,k}^{(j)}\right)^\intercal + \boldsymbol{S}_{1,k}^{(j)}\right] - \bar{\boldsymbol{b}}_j\bar{\boldsymbol{b}}_j^\intercal$
    Store $w_{1,1}^{(j)}, \ldots, w_{1,K}^{(j)}$ in the jth row of $\boldsymbol{W}$
    Include variable j in "expected residuals", $\boldsymbol{X}^\intercal\bar{\boldsymbol{R}} \leftarrow \boldsymbol{X}^\intercal\bar{\boldsymbol{R}}_{(j)} - \boldsymbol{X}^\intercal\boldsymbol{x}_j\bar{\boldsymbol{b}}_j^\intercal$
  Update prior weights, $\boldsymbol{w}_0 \leftarrow$ Update-Weights($\boldsymbol{W}$) ▷ See (16).
  Update residual covariance, $\boldsymbol{V} \leftarrow$ Update-Resid-Cov-ss($\boldsymbol{X}^\intercal\boldsymbol{X}, \boldsymbol{X}^\intercal\boldsymbol{Y}, \boldsymbol{Y}^\intercal\boldsymbol{Y}, \bar{\boldsymbol{B}}, \boldsymbol{S}_1, \ldots, \boldsymbol{S}_p$) ▷ See (20).
  $\text{ELBO}^{(t)} \leftarrow F(q; \boldsymbol{V}, \boldsymbol{w}_0)$
  $\delta \leftarrow \text{ELBO}^{(t)} - \text{ELBO}^{(t-1)}$
return $\bar{\boldsymbol{B}}$, $\boldsymbol{V}$, $\boldsymbol{w}_0$, $\text{ELBO}^{(t)}$

3. *mr.mash-rss* algorithm

Algorithms 1 and 2 provide pseudo-code to describe the fitting of the *mr.mash-rss* model. Algorithm 1 describes the variational algorithm to update the parameters $\boldsymbol{B}, \boldsymbol{V}$, and $\boldsymbol{w}_0$ using sufficient statistics.

Algorithm 2 describes the *mr.mash-rss* algorithm, which takes as input the summary statistics and includes the calculation of sufficient statistics and the subsequent estimation of the parameters via Algorithm 1. The summary statistics required, which are obtained from univariate simple linear regression models, are defined below.

- $\widehat{\boldsymbol{B}}$ is the $p \times r$ matrix whose j, s^{th} element is the least-squares estimate of the slope of the regression of $\boldsymbol{y}_s$ on $\boldsymbol{x}_j$. Its jth row is denoted by $\widehat{\boldsymbol{b}}_j^\intercal$.

- $\widehat{\boldsymbol{\Sigma}}_{\widehat{b}}$ is the $p \times r$ matrix whose j, s^{th} element is the estimated standard error of the estimated slope of the regression of $\boldsymbol{y}_s$ on $\boldsymbol{x}_j$. Its jth row is denoted by $\widehat{\boldsymbol{\sigma}}_{\widehat{b},j}^\intercal$.

- $\widehat{\boldsymbol{\Gamma}}$ is the $p \times p$ sample correlation matrix of $\boldsymbol{X}$.

The algorithm also requires $\boldsymbol{Y}^\intercal\boldsymbol{Y}$ (if available) and n.

†Operations inside this loop are elementwise multiplication and division.
‡This step calculates the PVE-adjusted z score. See [22] for details.

Algorithm 2 *mr.mash-rss*

Require: $\widehat{\boldsymbol{B}}$, $\widehat{\boldsymbol{\Sigma}}_{\widehat{b}}$, $\widehat{\boldsymbol{\Gamma}}$, $\boldsymbol{Y}^\intercal\boldsymbol{Y}$, n. Let $\boldsymbol{v}_Y = (\boldsymbol{y}_1^\intercal\boldsymbol{y}_1, \ldots, \boldsymbol{y}_r^\intercal\boldsymbol{y}_r)/(n-1)$ denote the $r-$vector containing the sample variances of each response.

Require: Set of K covariance matrices, $\mathscr{S}_0$.

Require: Initial estimates of the posterior mean coefficients, stored as a $p \times r$ matrix, $\bar{\boldsymbol{B}}$ whose j^{th} row is $\bar{\boldsymbol{b}}_j^\intercal$.

Require: Initial estimates of the prior mixture weights, $\boldsymbol{w}_0 = (w_{0,1}, \ldots, w_{0,K})$, and the $r \times r$ residual covariance matrix $\boldsymbol{V}$.

Require: Convergence threshold, tol ≥ 0, and an upper limit on the number of iterations, $t_{\max}$.

for j in $1, \ldots, p$ **do**†

  $\widehat{\boldsymbol{z}}_j := \widehat{\boldsymbol{b}}_j / \widehat{\boldsymbol{\sigma}}_{\widehat{b},j}$

  $\boldsymbol{a}_j := (n-1)/(n-2+\widehat{\boldsymbol{z}}_j^2)$

  $\tilde{\boldsymbol{z}}_j := \sqrt{\boldsymbol{a}_j}\widehat{\boldsymbol{z}}_j$ ‡

  $d_j := mean((\boldsymbol{v}_Y \boldsymbol{a}_j / \widehat{\boldsymbol{s}}_j^2))$

  $\boldsymbol{x}_j^\intercal\boldsymbol{Y} = \sqrt{\boldsymbol{a}_j}\tilde{\boldsymbol{z}}_j\boldsymbol{v}_y/\widehat{\boldsymbol{s}}_j$

$\boldsymbol{D}_x := \text{diag}(d_1, \ldots, d_p)$

$\boldsymbol{X}^\intercal\boldsymbol{X} = \boldsymbol{D}_x^{1/2}\widehat{\boldsymbol{\Gamma}}\boldsymbol{D}_x^{1/2}$

$\boldsymbol{X}^\intercal\boldsymbol{Y} = \begin{bmatrix} \boldsymbol{x}_1^\intercal\boldsymbol{Y} \\ \vdots \\ \boldsymbol{x}_p^\intercal\boldsymbol{Y} \end{bmatrix}$

  return $\boldsymbol{X}^\intercal\boldsymbol{X}$, $\boldsymbol{X}^\intercal\boldsymbol{Y}$, $\boldsymbol{Y}^\intercal\boldsymbol{Y}$.

$\bar{\boldsymbol{B}}$, $\boldsymbol{V}$, $\boldsymbol{w}_0$, ELBO$^{(t)}$ $\leftarrow$ mr.mash-sufficient($\boldsymbol{X}^\intercal\boldsymbol{X}$, $\boldsymbol{X}^\intercal\boldsymbol{Y}$, $\boldsymbol{Y}^\intercal\boldsymbol{Y}$, n, $\mathscr{S}_0$, $\boldsymbol{w}_0$, $\boldsymbol{V}$, $\bar{\boldsymbol{B}}$, tol, t_{max}). See Algorithm 1.

4. Details of the simulations

4.1. Data preparation

For the simulation analyses, we used real genotypes from the array data from the UK Biobank [3]. We kept individuals of Caucasian ancestry that self identified as White British. We computed relationships for every pairs of individuals based on genetic variants with minor allele frequency (MAF) greater than 0.01, minor allele count (MAC) greater than 5, Hardy-Weinberg equilibrium (HWE) test p-value greater than 10^{-10}, and missing genotype rate smaller than 0.1. We retained individuals such that no pair had relationship coefficient greater than 0.025, for a total of 251,288 individuals. We randomly sampled 105,000 individuals and filtered out genetic variants that did not pass the same filters as above in this subset. These steps were performed using PLINK (v. 1.90b7) [4]. Our final dataset for subsequent analyses included 595,071 genetic variants. Missing genotypes were imputed with the mean genotype for the respective genetic variant.

4.2. Simulation of phenotypes

For each replicate, we simulated the $n \times r$ matrix of phenotypes for $n = 105,000$ and $r = 5$ (or 10 in one simulation scenario) following the multivariate regression model in (5). The predictor matrix $\boldsymbol{X}$ was the filtered genotype data from the previous step (that was centered so that its columns have means equal to 0) and the effect matrix $\boldsymbol{B}$ was chosen to obtain the desired pattern of effect sharing. In particular, the effect, $\boldsymbol{b}_j$, of each causal variant across phenotypes was sampled from a distribution that depends on the scenario:

- "Equal Effects", "Low h_g^2", "High Polygenicity", "More Phenotypes", where each causal variant affects all the phenotypes and has the same effect across phenotypes. $\boldsymbol{b}_j \sim N_r(\boldsymbol{0}, \boldsymbol{1}_r\boldsymbol{1}_r^\intercal)$, where $\boldsymbol{1}_r = (1_1, \ldots, 1_r)^\intercal$.
- "Mostly Null", where the causal variants affect only the first phenotype while the remaining

phenotypes are affected only by a non-genetic component. $\boldsymbol{b}_j \sim N_r(\mathbf{0}, \boldsymbol{S})$, where $\boldsymbol{S}$ is an $r \times r$ matrix of all zeros except for a single one, $s_{11} = 1$.

- "Shared Effects in Subgroups", where the effect of each causal variant is drawn such that it is equally likely to be shared (but not be equal) in phenotypes 1 through 3 or to be shared (but not be equal) in phenotypes 4 and 5. $\boldsymbol{b}_j \sim 0.5 \times N_r(\mathbf{0}, \boldsymbol{S}_1) + 0.5 \times N_r(\mathbf{0}, \boldsymbol{S}_2)$ where $\boldsymbol{S}_1$ is an $r \times r$ matrix with diagonal elements equal to 1 and off-diagonal elements equal to 0.9, and $\boldsymbol{S}_2$ is an $r \times r$ matrix with diagonal elements equal to 1 and off-diagonal elements equal to 0.7.

The effects of the non-causal variants were set to 0. The residual covariance $\boldsymbol{V}$ was a diagonal matrix where the diagonal elements were chosen to obtain the desired genomic heritability. This implies that the residuals were uncorrelated across phenotypes. In the "Mostly Null" scenario, the diagonal elements for the phenotypes unaffected by genotypes were set to 1.

Finally, we randomly split the data (*i.e.*, $\boldsymbol{X}$ and $\boldsymbol{Y}$) into a training set (including 100,000 individuals) and a test set (including 5,000 individuals). The simulation procedure was repeated 20 times to obtain 20 replicates.

In the scenario with missing phenotypes, we assigned missing values completely at random (MCAR) to the phenotype matrix for the individuals in the training set. This was done such that each individual had missing values in any possible configuration of the five phenotypes with equal probability. This was done using `ampute` function from the R package mice (v. 3.16.0). We simulated two scenarios, one where 20% of the individuals had missing phenotypes and another one where 80% of the individuals had missing phenotypes, both based on the Equal Effects scenario.

4.3. Computation of the summary statistics and LD matrices

We computed the summary statistics using only individuals in the training set by performing a univariate linear regression of each quantile normalized phenotype on each genetic variant, one at a time, using the `big_univLinReg` function from the R package bigstatsr (v.1.5.12) [12]. The effect sizes and their standard errors were used for subsequent analyses. In the external LD scenario, we also performed strict quality control on the summary statistics as recommended in [14], following `https://privefl.github.io/bigsnpr-extdoc/polygenic-scores-pgs.html`. Briefly, this involved removing genetic variants whose frequency and standard deviation inferred from the summary statistics did not match with the ones in the external data used to compute the LD matrices.

The linkage disequilibrium matrices were computed using 146,288 nominally unrelated individuals ($r_a < 0.025$ between any pair) individuals of European ancestry (*i.e.*, Caucasian and white British fields), that did not overlap with the 105,000 individuals used to compute the summary statistics and assess prediction accuracy. This procedure resulted in a set of "out-of-sample" LD matrices. We used the function `snp_cor` from the R package bigsnpr (v.1.12.2) [12] to compute correlations between variants for each chromosome separately, setting the correlation between variants that are 3 cM or more apart to be 0, as previously recommended. This procedure resulted in "banded" LD matrices. [13].

To evaluate the robustness of *mr.mash-rss* to a truly "external" LD matrix, we computed LD matrices using 1000 Genomes project genotype data [6]. We extracted 503 European individuals and genetic variants that were present in our summary statistics and had MAF ≥ 0.05 in this subset of the 1000G data. We computed block-diagonal LD matrices, with the blocks defined in [1]. The correlation matrix for each block was "denoised" using its eigen decomposition and discarding eigenvalues smaller than 0.106 as suggested in [17].

4.4. Computation of genetic predictors

We compared five different methods for polygenic prediction:

- *LDpred2-auto* [13]. This is a univariate Bayesian linear regression model fit by Gibbs sampling, where the prior on the effect size of each genetic variant is:

$$b_j \sim (1-\pi)\delta_0 + \pi N(0, \frac{h_g^2}{p}) \tag{24}$$

We used the implementation in the R package bigsnpr (v.1.12.2) of this model. In particular, we used the `snp_ldpred2_auto` function that allows for the estimation of the hyperparameters (*i.e.*, π and h_g^2) from the data. We ran *LDpred2-auto* 30 times with a sequence of 30 values equally spaced on a log scale from 10^{-4} to 1 as initial values of π, and an estimate from LD Score regression (*LDSC*; obtained using the function `snp_ldsc`) as initial value of h_g^2. In each run, we set the number of burn-in iterations to 500, the number of iterations (after burn-in) to 1,000, and kept the other arguments as their default values. We obtained a final estimate of the effect size for each variant as the average of the effect sizes from the 30 runs, after discarding the chains that diverged, as recommended by the *LDpred2-auto* authors [13].

- *SBayesR* [9]. This is a univariate Bayesian linear regression model fit by Gibbs sampling, where the prior on the effect size of each genetic variant is:

$$b_j \sim \pi_0 \delta_0 + \sum_{k=1}^{K} \pi_k N(0, \gamma_k \sigma_b^2) \tag{25}$$

Here, we chose $K = 3$ and $\boldsymbol{\gamma} = (0.01, 0.1, 1.0)$. We used the implementation in the R package qgg (v. 1.1.2) [16] of this model. In particular, we used the `sblr` function that allows for the estimation of the hyperparameters (*i.e.*, $\boldsymbol{\pi}$ and σ_b^2) from the data. We initialized $h_g^2 = 0.1$ (which is used internally to initialize σ_b^2), we set the number of burn-in iterations to 1,000, the number of iterations to 5,000, the thinning parameter to 5, and kept the other arguments as their default values.

- *SmvBayesC* [5]. This is a multivariate Bayesian linear regression model fit by Gibbs sampling, where the prior on the effect size of each genetic variant is:

$$\begin{aligned} \boldsymbol{b}_j &= \boldsymbol{D}_j \boldsymbol{\beta}_j \\ \boldsymbol{\beta}_j &\sim N_r(\mathbf{0}, \boldsymbol{G}) \end{aligned} \tag{26}$$

where:

$$\boldsymbol{D} = \begin{bmatrix} d_{j1} & & \\ & \ddots & \\ & & d_{jr} \end{bmatrix}; \boldsymbol{G} = \begin{bmatrix} \sigma_{\beta 1}^2 & \cdots & \sigma_{\beta 1r} \\ \vdots & \ddots & \vdots \\ \sigma_{\beta 1r} & \cdots & \sigma_{\beta r}^2 \end{bmatrix}$$

$d_{js} \in 0, 1$ indicates whether variant j has an effect on phenotype s. Each potential configuration of effect presence/absence across phenotypes – $\boldsymbol{d}_{jl}$ for $l = 1, 2, \ldots, L = 2^r$ – is assigned a prior probability Π_l (subject to $\sum_{l=1}^{L} \Pi_l = 1$). $\boldsymbol{\Pi} = (\Pi_1, \Pi_2, \ldots, \Pi_L) \sim Dir(\boldsymbol{\alpha})$. We used the implementation in the R package qgg (v. 1.1.2) of this model. In particular, we used the `mtsblr` function that allows for the estimation of the hyperparameters (*i.e.*, $\boldsymbol{D}$ and $\boldsymbol{G}$) from the data. We initialized $h_g^2 = 0.1$ (which is used internally to initialize $\boldsymbol{G}$) and π – the probability of a genetic variant having an effect on at least one phenotype – as 0.0001 in every scenario, except in the "High Polygenicity" scenario where it was 0.01. We set the number of burn-in iterations to 1,000, the number of iterations to 5,000, the thinning parameter to 5, and we kept the other arguments as their default values.

- *SmvBayesC*-rest [7]. This is a multivariate Bayesian linear regression model similar to *SmvBayesC*, with the difference being that only 2 configurations for $\boldsymbol{d}_{jl}$ are allowed – an effect is either present in all or none of the phenotypes. We used the implementation in the R package qgg (v. 1.1.2) of this model. In particular, we used the `mtsblr` function that allows for the estimation of the hyperparameters (*i.e.*, $\boldsymbol{D}$ and $\boldsymbol{G}$) from the data. We initialized $h_g^2 = 0.1$ (which is used internally to initialize $\boldsymbol{G}$) and π – the probability of a genetic variant having an effect on all the phenotypes – as 0.0001 in every scenario, except in "High Polygenicity" where it was 0.01. We set the number of burn-in iterations to 1,000, the number of iterations to 5,000, the thinning parameter to 5, and kept the other arguments as their default values.

- *mr.mash-rss*. This is the method introduced in this paper and described extensively above and the main text. We used the function `mr.mash.rss` implemented in the R package mr.mash.alpha (v. 0.3.32). We initialized the posterior mean of the regression coefficients, $\bar{\boldsymbol{B}}$, to 0. We computed an initial estimate of the residual covariance, $\boldsymbol{V}$, from summary statistics by following a similar approach to [23]. For each chromosome, we selected genetic variants whose z-score was less than 2 (in absolute value) in every trait. We computed an initial estimate of $\boldsymbol{V}$ using the selected genetic variants for all chromosomes as:

$$\boldsymbol{V} = \frac{1}{J} \sum_{j=1}^{J} \boldsymbol{z}_j \boldsymbol{z}_j^{\intercal} \tag{27}$$

where J is the number of selected variants and $\boldsymbol{z}$ is an r-vector of z-scores across phenotypes. The mixture weights, $\boldsymbol{w}_0$, were initialized as 0.99 on $w_{0,0}$ (*i.e.*, the weight for the null component) and 0.01 split equally among the other mixture components. Both $\boldsymbol{V}$ and $\boldsymbol{w}_0$ were updated in the *mr.mash-rss* model fitting. $\boldsymbol{Y}^{\intercal}\boldsymbol{Y}$ was computed from the individual-level phenotypic data using only training individuals after quantile normalization. The algorithm was run until the difference in ELBO between two successive iterations was smaller than 0.01. To reduce runtime, at each iteration after the first 15 iterations, we dropped mixture components with estimated weight smaller than 10^{-8}. Following [11], we used an expanded version of the mixture prior:

$$\boldsymbol{b}_j \mid \boldsymbol{w}_0, \boldsymbol{\omega}, \mathscr{U}_0 \sim w_{0,0}\delta_{\boldsymbol{0}} + \sum_{l=1}^{L} \sum_{t=1}^{T} w_{0,l,t} N_r(\boldsymbol{0}, \omega_l^2 \boldsymbol{U}_{0,t}), \tag{28}$$

where $\delta_{\boldsymbol{0}}$ is the delta mass function at zero, $\boldsymbol{\omega}$ is a vector of scaling factors meant to capture the magnitude of the effect sizes and chosen as described in [19], and $\mathscr{U}_0$ is a list of normalized (such that the largest diagonal element was 1) covariance matrices meant to capture the patterns of effect sharing and specificity across phenotypes. The covariance matrices included "canonical" matrices:

  - The identity matrix, $\boldsymbol{I}_r$.
  - A matrix of all ones, $\boldsymbol{A} = \boldsymbol{1}_r \boldsymbol{1}_r^{\intercal}$, where $\boldsymbol{1}_r = (1_1, \ldots, 1_r)^{\intercal}$.
  - r rank-1 matrices of the form, $\boldsymbol{C}_s = \boldsymbol{c}_s \boldsymbol{c}_s^{\intercal}$, where $\boldsymbol{c}_s$ in an r-vector of all zeros except for a 1 at position s.
  - Three matrices with diagonal elements equal to 1 and off-diagonal elements equal to σ, with $\sigma = 0.25, 0.5, 0.75$, respectively.

  However, we also used "data-driven" matrices computed using the summary statistics for strong signals. In particular, for each chromosome, we selected genetic variants with z-score greater than 3 (in absolute value) in at least one phenotype. Then, we combined the selected genetic variants across chromosomes (m) to form the $m \times r$ matrix $\boldsymbol{Z}$.

  - Three rank-1 matrices based on the top 3 principal components of $\boldsymbol{Z}$, such that $\boldsymbol{P}_e = \boldsymbol{v}_e \boldsymbol{v}_e^{\intercal}$ where $\boldsymbol{v}_e$ is the eth right singular vector, for $e = 1, 2, 3$.
  - A rank-3 matrix based on the linear combination of the top 3 principal components of $\boldsymbol{Z}$, such that $\boldsymbol{P} = \frac{1}{m} \sum_{e=1}^{3} \sigma_e^2 \boldsymbol{v}_e \boldsymbol{v}_e^{\intercal}$ where σ_e^2 is the eth squared singular value.
  - A matrix based on the Empirical Bayes Matrix Factorization (EBMF) of $\boldsymbol{Z}$ as implemented in the R package flashier (v. 0.2.34). This is a matrix factorization method that produces a sparse low-rank approximation of the original matrix, while automatically selecting the rank of the approximation in a data-adaptive way [21]. $\boldsymbol{Q} = \frac{1}{m} \boldsymbol{F} \boldsymbol{L}^{\intercal} \boldsymbol{L} \boldsymbol{F}^{\intercal}$ where $\boldsymbol{L}$ is a matrix of estimated loadings and $\boldsymbol{L}$ is a matrix of estimated factors.
  - A number of matrices such that $\boldsymbol{Q}_e = \boldsymbol{f}_e \boldsymbol{f}_e^{\intercal}$ where f_e is the eth factor estimated by EBMF.

We then estimated the covariance matrices to use in *mr.mash-rss* by applying Extreme Deconvolution (ED) – implemented in the `cov_ed` function of the R package mashr (v. 0.2.79) – using the list of matrices above as initial estimates. In brief, this is an EM algorithm for fitting mixture models such as (6) and outputs a list of "denoised" covariance matrices [2]. This approach to estimate covariance matrices was introduced in [19] and has been used successfully in a few studies [11, 23].

- *MTAG+LDpred2-auto*. This is a two-step approach where MTAG was used in the first step. MTAG is a generalized method of moments estimator [18]:

$$\hat{\beta}_{MTAG,j,s} = \frac{\frac{\boldsymbol{\omega}_s^\intercal}{\omega_{ss}}(\Omega - \frac{\boldsymbol{\omega}_s\boldsymbol{\omega}_s^\intercal}{\omega_{ss}} + \Sigma_j)^{-1}}{\frac{\boldsymbol{\omega}_s^\intercal}{\omega_{ss}}(\Omega - \frac{\boldsymbol{\omega}_s\boldsymbol{\omega}_s^\intercal}{\omega_{ss}} + \Sigma_j)^{-1}\frac{\boldsymbol{\omega}_s}{\omega_{ss}}}\hat{\boldsymbol{\beta}}_j \tag{29}$$

where Ω is the covariance matrix of the true effect size across phenotypes and Σ is the covariance matrix of the residuals across phenotypes. These matrices are estimated from the summary statistics as explained in [18]. We used the implementation in the python package mtag (v. 1.0.8) with the following custom flags "–incld_ambig_snps" and "–median_z_cutoff 0.3". The LD scores were computed using the same UK Biobank individuals (or 1000G data, in the External LD scenario) as used to compute the LD matrices. This was done using the python package ldsc (v. 1.0.1) with 1 cM radius. Because MTAG does not deal with indels, we used the original OLS estimates for these genetic variants for the next step. The second step involved fitting *LDpred2-auto* using the MTAG-adjusted summary statistics. We fitted *LDpred2-auto* as described above, except for a few differences. First, we used the "robust" version of the method which involved setting the parameters "shrink_corr=0.95" and "allow_jump_sign=FALSE". Second, we computed an effective sample size as the weighted median of the ratio $\frac{\chi^2_{MTAG}}{\chi^2_{OLS}}$ for variants with $\chi^2_{OLS} > 30$.

- *wMT-SBLUP* [10]. This is a two-step approach that involves converting the univariate OLS summary statistics to univariate BLUP estimates using SBLUP in the first step. This was done using the implementation in the R package SumTool (v. 1.0.0) of this model. In particular, we used the `SBLUP` function with the shrinkage parameter $\lambda = p(\frac{1}{h^2} - 1)$ (with h^2 estimated using LDSC) and a window size of 2 Mb. The second step combines the BLUP estimates across phenotypes using selection index theory:

$$\hat{\beta}_{wMT-SBLUP,j,s} = \boldsymbol{w}^\intercal_{SBLUP,j}\hat{\boldsymbol{\beta}}_{SBLUP,j} \tag{30}$$

where:

$$\boldsymbol{w}_{SBLUP} = \boldsymbol{V}^{-1}_{SBLUP}\boldsymbol{C}_{SBLUP} \tag{31}$$

where $\boldsymbol{V}_{SBLUP}$ is the covariance matrix of the $\hat{\boldsymbol{\beta}}_{SBLUP}$ across phenotypes and $\boldsymbol{C}_{SBLUP}$ is a vector of the covariance between the true effects for the sth trait and $\hat{\boldsymbol{\beta}}_{SBLUP}$ for each of the other phenotypes. These quantities can be approximated using estimates of heritability and genetic correlations, and the number of independent genetic variants. The second step was done using the implementation in the R package qgg (v. 1.1.2) of this model. In particular, we used the `mtadj` function with estimates of the heritability of the phenotypes and the genetic correlations across phenotypes from LDSC and the number of effectively independent variants set to 60,000 [10].

Each method was applied to each chromosome separately. Phenotypic values for the test set individuals for a given phenotype s were predicted as:

$$\hat{\boldsymbol{y}}_{\text{test},s} = \sum_{g=1}^{22} \boldsymbol{X}_{\text{test},g}\hat{\boldsymbol{b}}_{g,s} \tag{32}$$

where $\boldsymbol{X}_{\text{test},g}$ is the genotype matrix including only genetic variants on the gth chromosome, and $\hat{\boldsymbol{b}}_{g,s}$ is a vector of estimated effect sizes of the genetic variants on the gth chromosome on the sth phenotype.

Prediction accuracy was evaluated as R^2 from the regression of $\boldsymbol{y}_{\text{test},s}$ on $\hat{\boldsymbol{y}}_{\text{test},s}$, where $\boldsymbol{y}_{\text{test},s}$ is a vector of actual (quantile normalized) phenotypic values for the test set individuals.

5. Details of the real data applications

5.1. Data preparation

For the real data application, we used 16 blood cell phenotypes from UK Biobank, that have been used in previous genetic analyses [20, 23]. As in [23], we focused on a subset of the individuals that met the following criteria:

- Were identified as White British by field 22006 in UK Biobank.
- No mismatch between self-reported and genetic sex.
- Were not outliers for missing genotype rate and/or heterozygosity as identified by field 22027 in UK Biobank.
- Did not have close relative as identified by field 22021 in UK Biobank.
- Were not pregnant.
- Did not have blood related disease based on ICD10 codes.
- Did not have any missing values.

Finally, after quantile normalization of each phenotype, we calculated the Mahalanobis distance for each individual ($\boldsymbol{y}_i^\intercal \hat{\Sigma}^{-1} \boldsymbol{y}_i$, where $\boldsymbol{y}_i$ is an r-vector of phenotypes for the ith individual and $\hat{\Sigma}$ is the sample phenotypic covariance matrix). We then excluded individuals falling above the 0.99 quantile of the χ^2_{16} distribution. After all the filtering, the final dataset had sample size of $n = 244,049$.

For the majority of the analyses, we used a set of $p = 1,054,330$ HapMap3 variants from the imputed UK Biobank genotype data. This set has been recommended in previous prediction analyses because the genetic variants included provide a good coverage of the genome and are well imputed [14].

Finally, we randomly assigned each individual to one of five subsets to perform 5-fold cross-validation. This consists of training each model on 4 out of the 5 subsets of the data (*i.e.*, the training set) and evaluating the model performance on the remaining subset (*i.e.*, the test set). This procedure is repeated 5 times, once for each subset.

For the second data application, we started from the n=244,049 individuals above and filtered out individuals that had missing values for any of the 8 additional traits. We adjusted the blood pressure values of individuals that reported taking blood pressure-lowering medications by adding 10 mmHg and 15 mmHg to diastolic pressure and systolic pressure, respectively [8]. Using the Mahalanobis distance-based approach above, we excluded individuals with outlying phenotypes. After these filters, the final dataset had sample size of $n = 216,739$.

5.2. Computation of the summary statistics and LD matrices

To compute summary statistics, we first adjusted each phenotype for the effect of sex, assessment center, age at recruitment, age $\times$ age, genotype measurement batch, and the first 10 genetic principal components by linear regression. The residuals for each phenotype were then quantile normalized and used as the response variable in a GWA analysis as described in Sec. 4.3. This procedure was performed 5 times, once for each training set in our 5-fold cross-validation scheme.

We computed a set of per-chromosome "banded" LD matrices for each training set based on the $n = 244,049$ individuals, using the same software and parameters as in Sec. 4.3. This procedure resulted in 5 sets of "in-sample" LD matrices for the blood cell traits analysis. The same matrices were also used for the second data application.

5.3. Computation of genetic predictors

We applied four different methods for polygenic prediction:

- *LDpred2-auto*. We used the same software and parameters as described in Sec. 4.4.
- *SBayesR*. We used the same software and parameters as described in Sec. 4.4.
- *mr.mash-rss*. We used the same software and a similar strategy to that described in Sec. 4.4, except for a few differences. First, we initialized the estimates of the regression coefficients with the estimates from *SBayesR*. *mr.mash-rss* solves a non-convex optimization problem and we found that a carefully chosen initialization could improve prediction accuracy [11]. Second, we computed the residual covariance in the same way as described above. However, we did not update it within the *mr.mash-rss* algorithm as that is the only step that requires an estimate of $\boldsymbol{Y}^\intercal\boldsymbol{Y}$, which might not always be available. Thus, we wanted to compare the methods in a realistic scenario. Third, we used a different strategy to select strong signals to compute the "data-driven" covariance matrices. For each training set:
  - We performed association analyses for each trait and all biallelic autosomal SNPs with MAF greater than 0.001, and INFO score greater than 0.6 using the procedure described in Sec. 5.2.
  - We obtained a list of 975 non-overlapping genomic regions that were used for fine-mapping the same 16 blood cell phenotypes in a previous study [23]. The same strategy resulted in 588 non-overlapping genomic regions for the second data application.
  - For each of the genomic regions, we fine-mapped the association results using the *SuSiE-RSS* method (implemented in the R package susieR (v. 0.12.40), with default parameters). For each phenotype, we selected genetic variants with the highest posterior inclusion probability (PIP) within each credible set (CS).
  - We combined the lists of selected genetic variants across phenotypes and genomic regions to obtain a final list of genetic variants with strong evidence of association. These genetic variants were then used to compute the "data-driven" covariance matrices as described in Sec. 4.4.
- *mr.mash-rss mash*. This is a combined strategy where we used *mash* to obtain good initial estimates of the mixture weights. In particular, for each chromosome separately, we obtained a set of approximately independent genetic variants by clumping based on MAF using the function `snp_clumping`) from the R package bigsnpr (v. 1.12.2), with an LD r^2 threshold of 0.01. We combined the list of approximately independent variants for all the chromosomes, to have enough genetic variants for the following step. We fit *mash* using the function `mash` from the R package mashr (v. 0.2.79) to the summary statistics for the LD-pruned genetic variants, using the same mixture prior and the same residual covariance as for *mr.mash-rss*. We extracted the estimated mixture weights, set the weight on the null component to 0.5, and rescaled the other mixture weights accordingly. These mixture weights were used as initial estimates for *mr.mash-rss*, which was then constrained to update the mixture weights for only the first 10 iterations, leaving them fixed for the remaining iterations until convergence of the algorithm.

5.4. Analysis with sampled individuals

We randomly sampled 15,000 individuals for each fold to obtain a total of 75,000 individuals. We repeated all the analyses described above, except that we did not recompute LD matrices and used the ones already computed for the full data analysis. Given the much smaller sample size, we also increased the MAF threshold to 0.01 for the association analysis used as part of the computation procedure for the *mr.mash-rss* prior.

6. Computing environment

All the analyses were run on Linux machines (Rocky Linux 8.5) with Intel Xeon Platinum 8380, Intel Xeon Gold 6348, or Intel Xeon Gold 6448H processors. We used R v. 4.2.3 [15] linked to Intel oneAPI Math Kernel Library (oneMKL) v. 2023.0.0.

References

[1] T. Berisa and J. K. Pickrell. Approximately independent linkage disequilibrium blocks in human populations. *Bioinformatics*, 32(2):283, 2016.

[2] J. Bovy, D. W. Hogg, and S. T. Roweis. Extreme Deconvolution: inferring complete distribution functions from noisy, heterogeneous and incomplete observations. *Annals of Applied Statistics*, 5 (2B):1657–1677, 2011.

[3] C. Bycroft, C. Freeman, D. Petkova, G. Band, L. T. Elliott, K. Sharp, A. Motyer, D. Vukcevic, O. Delaneau, J. O'Connell, et al. The uk biobank resource with deep phenotyping and genomic data. *Nature*, 562(7726):203–209, 2018.

[4] C. C. Chang, C. C. Chow, L. C. Tellier, S. Vattikuti, S. M. Purcell, and J. J. Lee. Second-generation plink: rising to the challenge of larger and richer datasets. *Gigascience*, 4(1):s13742–015, 2015.

[5] H. Cheng, K. Kizilkaya, J. Zeng, D. Garrick, and R. Fernando. Genomic prediction from multiple-trait bayesian regression methods using mixture priors. *Genetics*, 209(1):89–103, 2018.

[6] . G. P. Consortium et al. A global reference for human genetic variation. *Nature*, 526(7571):68, 2015.

[7] Y. Jia and J.-L. Jannink. Multiple-trait genomic selection methods increase genetic value prediction accuracy. *Genetics*, 192(4):1513–1522, 2012.

[8] M. Kerin and J. Marchini. Inferring gene-by-environment interactions with a bayesian whole-genome regression model. *The American Journal of Human Genetics*, 107(4):698–713, 2020.

[9] L. R. Lloyd-Jones, J. Zeng, J. Sidorenko, L. Yengo, G. Moser, K. E. Kemper, H. Wang, Z. Zheng, R. Magi, T. Esko, et al. Improved polygenic prediction by bayesian multiple regression on summary statistics. *Nature communications*, 10(1):5086, 2019.

[10] R. M. Maier, Z. Zhu, S. H. Lee, M. Trzaskowski, D. M. Ruderfer, E. A. Stahl, S. Ripke, N. R. Wray, J. Yang, P. M. Visscher, et al. Improving genetic prediction by leveraging genetic correlations among human diseases and traits. *Nature communications*, 9(1):989, 2018.

[11] F. Morgante, P. Carbonetto, G. Wang, Y. Zou, A. Sarkar, and M. Stephens. A flexible empirical bayes approach to multivariate multiple regression, and its improved accuracy in predicting multi-tissue gene expression from genotypes. *PLoS Genetics*, 19(7):e1010539, 2023.

[12] F. Privé, H. Aschard, A. Ziyatdinov, and M. G. Blum. Efficient analysis of large-scale genome-wide data with two r packages: bigstatsr and bigsnpr. *Bioinformatics*, 34(16):2781–2787, 2018.

[13] F. Privé, J. Arbel, and B. J. Vilhjálmsson. Ldpred2: better, faster, stronger. *Bioinformatics*, 36 (22-23):5424–5431, 2020.

[14] F. Privé, J. Arbel, H. Aschard, and B. J. Vilhjálmsson. Identifying and correcting for misspecifications in gwas summary statistics and polygenic scores. *Human Genetics and Genomics Advances*, 3(4), 2022.

[15] R Core Team. *R: A Language and Environment for Statistical Computing.* R Foundation for Statistical Computing, Vienna, Austria, 2023. URL `https://www.R-project.org/`.

[16] P. D. Rohde, I. Fourie Sørensen, and P. Sørensen. Expanded utility of the r package, qgg, with applications within genomic medicine. *Bioinformatics*, 39(11):btad656, 2023.

[17] J. P. Spence, N. Sinnott-Armstrong, T. L. Assimes, and J. K. Pritchard. A flexible modeling and inference framework for estimating variant effect sizes from gwas summary statistics. *BioRxiv*, pages 2022–04, 2022.

[18] P. Turley, R. K. Walters, O. Maghzian, A. Okbay, J. J. Lee, M. A. Fontana, T. A. Nguyen-Viet, R. Wedow, M. Zacher, N. A. Furlotte, et al. Multi-trait analysis of genome-wide association summary statistics using mtag. *Nature genetics*, 50(2):229–237, 2018.

[19] S. M. Urbut, G. Wang, P. Carbonetto, and M. Stephens. Flexible statistical methods for estimating and testing effects in genomic studies with multiple conditions. *Nature genetics*, 51(1):187–195, 2019.

[20] D. Vuckovic, E. L. Bao, P. Akbari, C. A. Lareau, A. Mousas, T. Jiang, M.-H. Chen, L. M. Raffield, M. Tardaguila, J. E. Huffman, et al. The polygenic and monogenic basis of blood traits and diseases. *Cell*, 182(5):1214–1231, 2020.

[21] W. Wang and M. Stephens. Empirical bayes matrix factorization. *Journal of Machine Learning Research*, 22(120):1–40, 2021.

[22] Y. Zou, P. Carbonetto, G. Wang, and M. Stephens. Fine-mapping from summary data with the “sum of single effects” model. *PLoS Genetics*, 18(7):e1010299, 2022.

[23] Y. Zou, P. Carbonetto, D. Xie, G. Wang, and M. Stephens. Fast and flexible joint fine-mapping of multiple traits via the sum of single effects model. *bioRxiv*, pages 2023–04, 2023.
