# Supplementary material for: Improving polygenic prediction from summary data by learning patterns of effect sharing across multiple phenotypes
Source: PLoS Genet. 2025 Jan 7;21(1):e1011519. doi: 10.1371/journal.pgen.1011519 (PMC11741642; doi:10.1371/journal.pgen.1011519)
Supplement: Attachment — Submitted filename: response_to_reviewers.pdf [file pgen.1011519.s008.pdf]

RESPONSE TO REVIEWS AND SUMMARY OF THE REVISIONS

By Deborah Kunkel[1], Peter Sørensen[2], Vijay Shankar[3], and Fabio Morgante[4]

[1]*School of Mathematical and Statistical Sciences, Clemson University, Clemson, SC, United States of America, dekunke@clemson.edu*

[2]*Center for Quantitative Genetics and Genomics, Aarhus University, Aarhus, Denmark*

[3]*Center for Human Genetics, Clemson University, Greenwood, SC, United States of America*

[4]*Center for Human Genetics, Clemson University, Greenwood, SC, United States of America, fabiom@clemson.edu*

We thank the reviewers for their feedback on our manuscript. We made several changes to the manuscript to address the reviewers' comments, which we summarize here:

1. We have included additional multivariate methods to our comparisons.
2. We have investigated the robustness of *mr.mash-rss* to some model misspecifications, namely the use of external LD matrices and the presence of non-overlapping samples.
3. We have added an additional real data application.

We have also highlighted in blue the parts of the main text that have changed substantially in the revised version.

Main comments from Reviewer 1.

Introduction. Thank you for the opportunity to review your manuscript titled "Improving polygenic prediction from summary data by learning patterns of effect sharing across multiple phenotypes." Your work on extending the mr.mash model to utilize summary statistics (*mr.mash-rss*) is a valuable contribution to the field of polygenic prediction. I have a few suggestions and comments that I hope you find helpful in refining your manuscript.

Our response. We thank the reviewer for taking the time to review our manuscript and for the nice words about our work.

Comment 1. Your proposed method, *mr.mash-rss*, shows significant similarities to existing approaches, particularly mr.mash and mashR. While extending mr.mash to use summary statistics is a valuable adaptation, it would be beneficial to clearly articulate how your work distinguishes itself from these established methods. Highlighting the unique contributions and specific advancements of *mr.mash-rss* will help emphasize its novelty and impact.

Our response. We thank the reviewer for this suggestion. We discussed the difference between *mr.mash* and *mr.mash-rss* (*i.e.*, the latter being the summary statistics extension of the former) in the abstract and at lines 23-42 of the main text. We have now added a brief comparison with *mash*, highlighting the main differences, in the introduction.

Comment 2. The incorporation of variational inference and empirical Bayes in *mr.mash-rss* is a noteworthy addition. However, the manuscript could benefit from a more detailed explanation of these techniques and their specific advantages in your context. Clarifying how variational inference improves computational efficiency and how empirical Bayes enhances adaptability will provide a clearer understanding of the strengths of your approach.

Our response. We thank the reviewer for this suggestion. The main contribution of the present work is to extend the *mr.mash* model to use only summary statistics. The use of a variational empirical Bayes (VEB) approach in multivariate regression was the main contribution of the work that introduced the *mr.mash* model. A detailed description of variational inference and empirical Bayes, including the advantages in this context, was given in that paper (Morgante et al., 2023). We feel that a detailed explanation of the VEB approach would "dilute" the main contribution of the present work. However, we included a sentence to direct the reader to Morgante et al. (2023) for details about the VEB approach.

Comment 3. The manuscript mentions that while $\boldsymbol{X}^\intercal \boldsymbol{Y}$ can be recovered exactly, $\boldsymbol{X}^\intercal \boldsymbol{X}$ is approximated using LD reference panels. It would be helpful to discuss the potential biases introduced by this approximation, especially when the reference panel does not perfectly match the study population.

Additionally, addressing the assumption that summary statistics are computed using the same individuals for each response will clarify the applicability of your method in real-world scenarios where missing data are common.

Our response. We thank the reviewer for this suggestion. Instead of a discussion, we have added simulations to investigate the robustness of the method to different proportion of missing values in the phenotype matrix (*i.e.*, fewer overlapping samples) and to completely out-of-sample LD using the 1000G data. The results showed that *mr.mash-rss* is robust to a small percentage of missing values. As the percentage of missing values increases, *mr.mash-rss*' performance decreases, although it remains better than the univariate methods. We also showed that *mr.mash-rss* is fairly robust to completely out-of-sample LD. We believe these additional investigations have strengthened our paper.

Comment 4. Including comparisons with a wider array of existing methods, such as different Bayesian approaches and multi-trait models, will provide a clearer context for the improvements claimed by *mr.mash-rss*. Evaluating your method across various datasets with different characteristics (e.g., sample sizes, ethnic backgrounds, trait architectures) will strengthen the evidence for its general applicability and robustness.

Our response. We thank the reviewer for this suggestion. We have added additional methods to the comparisons (*i.e.*, wMT-SBLUP and MTAG+LDpred2) and have added another data application with more polygenic traits. Our initial submission had already examined the effect of sample size on the prediction accuracy of *mr.mash-rss* in the blood cell traits analysis, and we decided that additional comparisons were unnecessary. While certainly interesting, applications to additional ethnic backgrounds are outside the scope of the current work.

Comment 5. Clearly articulating the unique contributions of *mr.mash-rss* and how they advance the field will enhance the manuscript. Detailed explanations of the innovations in model design and the practical impact of these methodological improvements will help readers appreciate the significance of your work.

Our response. Please see the response to Comments 1 and 2.

Comment 6. It might be valuable to explore whether a simpler approach using mashR could be effective. For instance, one could analytically compute summary statistics and then apply shrinkage using the LD matrix. This could offer a more straightforward and computationally efficient solution while still leveraging the benefits of LD information.

Our response. We thank the reviewer for this suggestion. While we agree that a modular approach using *mash* to adjust univariate summary statistics according to patterns of sharing across traits, followed by a univariate regression method (e.g., LDpred2) for each trait has

value, we believe that our combined approach in *mr.mash-rss* has some important advantages. First, it is easier to use because it involves only one step. Second, *mr.mash-rss* uses all the variants to estimate the mixture weights. On the other hand, *mash* assumes that genetic variants are independent of each other. That means that to estimate the mixture weights, the user needs to pre-select genetic variants using some sort of LD pruning. This procedure could result in biased estimates of the mixture weights, in addition to adding an additional step to the workflow. Third, *mr.mash-rss* takes full account of sample overlap by fitting a residual covariance across phenotypes. The modular approach takes account of sample overlap only in the *mash* step, but not in the following univariate regression. Finally, all the multiple regression methods with summary statistics (e.g., LDpred2 and SBayesR) assume that the summary statistics come from simple ordinary least squares regression. When summary statistics from other methods such as linear mixed models or MTAG are used, the multiple regression methods tend to have convergence issues (we experienced that first hand in our simulations using MTAG+LDpred2). This could potentially be worse when using summary statistics from *mash* since these are posterior means and standard deviations from a Bayesian method.

Having said that, this comment inspired us in the use of *mash*-estimated mixture weights to improve *mr.mash-rss*' performance with more polygenic traits. Thank you!

Other comments from Reviewer 1.

- Provide more detailed insights into the implementation and advantages of variational inference in *mr.mash-rss*.
- Discuss the empirical Bayes techniques used and compare them with other adaptive methods.
- Acknowledge and discuss the limitations introduced by the approximation of $\boldsymbol{X}^\intercal \boldsymbol{X}$ and the assumptions made, particularly in diverse population contexts.
- Include a wider range of benchmarks to contextualize the improvements and demonstrate the practical significance of *mr.mash-rss*.

Our response. We thank the reviewer for these suggestions. We have provided detailed responses in the previous sections.

Main comments from Reviewer 2.

Introduction. The authors have demonstrated that *mr.mash-rss* is a novel to learn pattern of shared effects across multiple phenotypes using GWAS summary statistics. The manuscript was well-written and clearly showed the purpose of the paper. While there is a major advancement in this work, there are several questions I would like to ask.

Our response. We thank the reviewer for taking the time to review our manuscript and for the positive words about our work.

Comment 1. In simulations, I wonder if the authors could simulate using the same HapMap3 variants as SBayesR and LDpred2 were designed with this set of SNPs.

Our response. We thank the reviewer for this suggestion. We decided to use the array data from UK Biobank in order to speed up the numerous simulations we performed. We believe that using HapMap3 variants would not change the conclusions of our simulations, but would significantly increase the run time because of the larger number of variants. We note that we did use HapMap3 variants in our real data applications.

Comment 2. On line 271-273, the author mentioned about the application to non-European descent, I would think this transferability is also due to the assumption of shared causal SNPs between ancestral groups.

Our response. We would like to clarify that the statement mentioned by the reviewer is not about transferability of predictions across ancestry groups. We simply meant that *mr.mash-rss* could be particularly useful within datasets of non-European individuals, because their sample size is usually smaller than those of European individuals. And we showed that the improvement in accuracy provided by *mr.mash-rss* is higher with smaller sample size (see blood cell traits application).

Comment 3. I wonder how sensitive the method to GWASes with fewer overlapping samples and out-of-sample LD matrices (e.g. LD from 1000 Genomes).

Our response. We thank the reviewer for this suggestion. We have added simulations to investigate the robustness of the method to missing values in the phenotype matrix (*i.e.*, fewer overlapping samples), and completely out-of-sample LD using the 1000G data. The results showed that *mr.mash-rss* is robust to a small percentage of missing values. As the percentage of missing values increases, *mr.mash-rss*' performance decreases, although it remains better than the univariate methods. We also showed that *mr.mash-rss* is fairly robust to completely out-of-sample LD. We also note that all the other simulations use "out-of-sample" LD in that the LD matrix was computed using a non-overlapping sample of UK Biobank individuals to that used to compute the summary statistics.

Comment 4. I wonder if the author can evaluate the method in an independent cohort to demonstrate the power of the methods.

Our response. We thank the reviewer for this suggestion. Unfortunately, we only have access to the UK Biobank data, so we are unable to evaluate the method in another cohort. However, we have added a second real data application using UK Biobank and additional traits.

Main comments from Reviewer 3.

Introduction. Kunkel et al. is a very nice paper that I really enjoyed reading. The method is concisely and clearly described, and the work is well organized. The paper extends the mr.mash method so that it can be fitted using only GWAS summary statistics, which if works, makes it much more useful in practice (as the authors note). Overall it's a very nice study, however I believe it has some potential limitations that could be addressed. Below I provide some suggestions aimed at improving the work, where the main concern is robustness of the method in real scenarios and applicability. Also, I think the benchmarks could be extended to consider other potentially more effective multivariate methods.

Our response. We thank the reviewer for taking the time to review our manuscript and the nice words about our work.

Comment 1. It seems to me that the method assumes that the covariance of genetic effects is known across outcomes ($\boldsymbol{S}_0$). Do you estimate this using LD score regression?

Also, it seems that the method also assumes $\boldsymbol{Y}^\intercal\boldsymbol{Y}$ is known. However, this may not be directly estimable in practice, but could be obtained using LD score regression. LD score does however make different assumptions.

I would be interested in seeing simulations and real data analyses that examined the performance when the GWAS summary stats are obtained on different cohorts, using partially and non-overlapping samples. Such analyses would in my mind represent the most useful applications scenarios for the method.

Our response. Regarding the covariance of genetic effects, *mr.mash-rss* assumes it to be known. While the user can specify their preferred covariance structure, in this manuscript, we used methods from Urbut et al. (2019), as we have found them to be effective. Specific details are available in section 4.4 (under the *mr.mash-rss* bullet point) of the S1 Text.

Regarding $\boldsymbol{Y}^\intercal\boldsymbol{Y}$, this quantity is strictly needed only if the user wants to update the residual covariance. We mention this at lines 96-98 of the main text.

We thank the reviewer for the suggestion of investigating the robustness of *mr.mash-rss* to non-overlapping samples. To do so, we added two simulation scenarios where we assigned missing values to the phenotype matrix for 20% and 80% of the individuals, respectively. The results showed that the performance of *mr.mash-rss* remains unaffected in the 20% scenario, while it deteriorates in the 80% scenario. However, even in this case the performance of *mr.mash-rss* is still better than the univariate methods. Thus, *mr.mash-rss* is fairly robust to a small-medium amount of missing values. We did not investigate the completely non-overlapping samples scenario. *mr.mash-rss* is not intended to be used in this case as it is a complete violation of the methods' assumptions.

Comment 2. SBayesR and LDpred1/2, and other iterative methods do not always converge in practice when the summary statistics are somehow imperfect. This is not only an LD reference panel issue, which is obviously important as you note. E.g., in meta-analyses sample sizes often vary between SNPs, as not all SNPs may be called or imputed accurately across all cohorts. Also, different cohorts in meta-analyses may be subject to different biases and noise levels. Some of these biases could be examined in simulations, e.g. varying sample sizes, and LD reference quality. However, the best test is using real external GWAS summary statistics. You can examine public single-cohort summary statistics such as FinnGen or decode genetics summary statistics and predict into UKB.

Our response. We agree with the reviewer that "imperfect" summary statistics are problematic. Using FinnGen or Decode summary statistics to train the model and then predict in UK Biobank would introduce additional complexities such as potential population differences in LD patterns and causal effects. These issues and the ones the reviewer mentions would all get confounded. Thus, in the revision, we addressed a couple of those issues in simulations, namely LD reference quality and different sample sizes among phenotypes. We believe that the investigation of different sample sizes among phenotypes indirectly addresses the issue of different sample sizes among SNPs, as both issues result in mismatches between the summary statistics and the LD matrix.

Comment 3. The paper doesn't examine applications to case-control outcomes. The methods that it compares against are generally all applicable to such outcomes (although the math involved generally assumes that they are quantitative). I see no clear reasons why mr.mash couldn't be applied to such outcomes, and please explain if that's not the case. Many disease outcomes are highly correlated, e.g. psychiatric disorders, cardiometabolic disorders, etc.

The real data considered all have relatively simple genetic architectures. I would also consider outcomes such as height, which could also be correlated to other anthropometric outcomes in the UKB. Or if possible, disease outcomes.

Our response. We thank the reviewer for these suggestions. We think that *mr.mash-rss* could be applied to binary outcomes, in the same way LDpred2 and SBayesR can. However, this application is outside the scope of the current work. We have now added a few sentences about this in the discussion.

We have included an additional data application including traits that are more polygenic. The results showed that *mr.mash-rss* is not as effective for those traits. This is a known issue for this type of variational empirical Bayes methods and is due the approximate M-step for updating the mixture weights (Zabad, Gravel and Li, 2023; Kim et al., 2024). We

have devised a combined strategy that uses *mash*-estimated mixture weights to improve the performance of *mr.mash-rss* with more polygenic traits. However, finding a more principled approach is left for future work. We have added a paragraph to the discussion highlighting this limitation of the method and the need for more work in this area.

Comment 4. There are a couple of multivariate methods that I would recommend also considering. First, there's SBLUP (Maier et al., 2018). I also recommend comparing with some of the following MTAG (Turley et al., 2018), GSEM (Grotzinger et al., 2019), or even multi-PGS (Albiñana et al., 2023). I do however concede that using some of these might be more complicated in practice as these are two-step approaches, in that you first have to generate multi-trait summary statistics and then fit using a single outcome PGS. However, I suspect that this could be more powerful in some settings, more generalizable and more robust. (I don't expect you to compare against all of these, but I recommend some.)

Our response. We thank the reviewer for this suggestion. We have added wMT-SBLUP and MTAG followed by LDpred2 (MTAG+LDpred2) to the simulations. The results showed that these methods are generally not as effective as *mr.mash-rss* and the other methods in the original comparison. In particular, the infinitesimal architecture assumption of wMT-SBLUP makes it unsuitable for all but the most polygenic traits and our results confirm that. MTAG+LDpred2's performance was generally similar to the univariate methods, except for the "Low h2g" and the "High Polygenicity" where it performed better. However, we did experience convergence issues with LDpred2 when using MTAG summary statistics, despite using the robust configuration of LDpred2 (Privé et al., 2022).

Comment 5. There is no run time comparison, but this would be highly useful for people when deciding whether to use the software if this was presented.

Our response. We thank the reviewer for this suggestion. We have added run time comparisons to S1 Table.

Comment 6. It wasn't clear to me exactly how the causal effects were sampled in the simulations. Did you sample their effects using equation 2? If so, what weights and values K did you use? Perhaps trying a couple combinations of these values would be of interest, to see how the performance varies with these.

Our response. A detailed description of how the causal effects were sampled in each scenario can be found in section 4.2 of the S1 Text. Briefly, effects were drawn from a zero-centered Normal distribution with covariance matrix chosen to achieve the desired patterns of sharing across phenotypes in all scenarios, except for the "Shared Effects in Subgroups". In that case, effects were drawn from a two-component mixture-of-multivariate Normals.

REFERENCES

ALBIÑANA, C., ZHU, Z., SCHORK, A. J., INGASON, A., ASCHARD, H., BRIKELL, I., BULIK, C. M., PETERSEN, L. V., AGERBO, E., GROVE, J. et al. (2023). Multi-PGS enhances polygenic prediction by combining 937 polygenic scores. *Nature communications* **14** 4702.

GROTZINGER, A. D., RHEMTULLA, M., DE VLAMING, R., RITCHIE, S. J., MALLARD, T. T., HILL, W. D., IP, H. F., MARIONI, R. E., MCINTOSH, A. M., DEARY, I. J. et al. (2019). Genomic structural equation modelling provides insights into the multivariate genetic architecture of complex traits. *Nature human behaviour* **3** 513–525.

KIM, Y., WANG, W., CARBONETTO, P. and STEPHENS, M. (2024). A flexible empirical Bayes approach to multiple linear regression and connections with penalized regression. *Journal of Machine Learning Research* **25** 1–59.

MAIER, R. M., ZHU, Z., LEE, S. H., TRZASKOWSKI, M., RUDERFER, D. M., STAHL, E. A., RIPKE, S., WRAY, N. R., YANG, J., VISSCHER, P. M. et al. (2018). Improving genetic prediction by leveraging genetic correlations among human diseases and traits. *Nature communications* **9** 989.

MORGANTE, F., CARBONETTO, P., WANG, G., ZOU, Y., SARKAR, A. and STEPHENS, M. (2023). A flexible empirical Bayes approach to multivariate multiple regression, and its improved accuracy in predicting multi-tissue gene expression from genotypes. *PLoS Genetics* **19** e1010539.

PRIVÉ, F., ARBEL, J., ASCHARD, H. and VILHJÁLMSSON, B. J. (2022). Identifying and correcting for mis-specifications in GWAS summary statistics and polygenic scores. *Human Genetics and Genomics Advances* **3**.

TURLEY, P., WALTERS, R. K., MAGHZIAN, O., OKBAY, A., LEE, J. J., FONTANA, M. A., NGUYEN-VIET, T. A., WEDOW, R., ZACHER, M., FURLOTTE, N. A. et al. (2018). Multi-trait analysis of genome-wide association summary statistics using MTAG. *Nature genetics* **50** 229–237.

URBUT, S. M., WANG, G., CARBONETTO, P. and STEPHENS, M. (2019). Flexible statistical methods for estimating and testing effects in genomic studies with multiple conditions. *Nature genetics* **51** 187–195.

ZABAD, S., GRAVEL, S. and LI, Y. (2023). Fast and accurate Bayesian polygenic risk modeling with variational inference. *The American Journal of Human Genetics* **110** 741–761.
