# Supplementary material for: Improving polygenic prediction from summary data by learning patterns of effect sharing across multiple phenotypes
Source: PLoS Genet. 2025 Jan 7;21(1):e1011519. doi: 10.1371/journal.pgen.1011519 (PMC11741642; doi:10.1371/journal.pgen.1011519)
Supplement: Attachment — Submitted filename: response_to_reviewers.pdf [file pgen.1011519.s009.pdf]

RESPONSE TO REVIEWS AND SUMMARY OF THE REVISIONS

By Deborah Kunkel[1], Peter Sørensen[2], Vijay Shankar[3], and Fabio Morgante[4]

[1]*School of Mathematical and Statistical Sciences, Clemson University, Clemson, SC, United States of America, dekunke@clemson.edu*

[2]*Center for Quantitative Genetics and Genomics, Aarhus University, Aarhus, Denmark*

[3]*Center for Human Genetics, Clemson University, Greenwood, SC, United States of America*

[4]*Center for Human Genetics, Clemson University, Greenwood, SC, United States of America, fabiom@clemson.edu*

We thank the reviewers for their feedback on our manuscript. We made several changes to the manuscript to address the reviewers' comments, which we summarize here:

1. We have included the citations of the integrative approaches suggested by Reviewer 2.
2. We have added a paragraph to the Discussion where we highlight the suggestion made by Reviewer 3 to evaluate the robustness of *mr.mash-rss* using truly external summary statistics.

We have also highlighted in blue the parts of the main text that have changed substantially in the revised version.

Main comments from Reviewer 1.

Introduction. All comments have been addressed.

Our response. We are pleased that the reviewer believes we addressed their comments. We thank the reviewer for their helpful suggestions that improved our manuscript.

Main comments from Reviewer 2.

Introduction. The authors nicely addressed all of my comments!

Our response. We are pleased that the reviewer believes we addressed their comments. We thank the reviewer for their helpful suggestions that improved our manuscript.

Comment 1. For integrative PRS, I would cite Albiñana et al. (2023) and Truong et al. (2024) because they recently proposed complementary methods to perform PRS combination.

Our response. We thank the reviewer for this suggestion. We have added those citations to the manuscript.

Main comments from Reviewer 3.

Introduction. Thank you for addressing many of my concerns and the comments made by other reviewers. I believe the manuscript has improved substantially, although I still have a couple of perhaps minor comments.

Our response. We are pleased that the reviewer believes we addressed their major comments. We thank the reviewer for their helpful suggestions that improved our manuscript.

Comment 1. On your response to comment 2, I still believe this would be the most fair comparison for a summary statistics based method, but perhaps you can highlight this in the discussion.

Our response. We thank the reviewer for this suggestion. We have added a paragraph to the Discussion articulating this point.

Comment 2. On your response to comment 3, I don't understand your reasoning for not applying mr.mash-rss to binary outcomes, especially because you also note that the methods that you compare against do. I suspect that these outcomes would be more polygenic than molecular measurements, and would therefore look like the ones in Supplementary Figure 3. Interestingly, the results in Supplementary Figure 3 suggest that mr.mash-rss underperforms on highly polygenic traits like BMI. It's ok if mr.mash-rss doesn't outperform other methods in every way, and I think exploring a few common case-control outcomes would be of interest.

Our response. We thank the reviewer for this suggestion. We do agree that the application of *mr.mash-rss* to binary outcomes would be of interest. However, we believe that a proper evaluation of the performance of *mr.mash-rss* on binary outcomes would require (1) an array of simulations similar to those we did for continuous outcomes; (2) an evaluation of whether to use summary statistics from linear regression (it satisfies *mr.mash-rss* ' assumptions, but it is less appropriate for binary traits) and logistic regression (it is appropriate for binary traits, but it violates *mr.mash-rss* ' assumptions); (3) a real data application. Thus, we believe that evaluating the performance of *mr.mash-rss* on binary outcomes is best addressed in additional future work and is not critical for the claims we make in the current manuscript. In fact, other polygenic prediction methods papers (*e.g.*, Zeng and Zhou (2017)) have focused on continuous outcomes only, mentioning the investigation of binary outcomes as a future direction in the discussion.

Comment 3. On comment 4, why didn't compare against MTAG+SBayesR instead of MTAG+LDpred2, as SBayesR seemed to outperform LDpred2?

Our response. We thank the reviewer for this comment. This choice was made based on the fact that SBayesR seemed less robust than LDpred2 to model misspecifications (see our results with external LD matrix). Since using summary statistics obtained from MTAG violates one of the assumptions made by most polygenic prediction methods (*i.e.*, that summary statistics are obtained by OLS regression), we opted for the more robust LDpred2.

REFERENCES

ALBIÑANA, C., ZHU, Z., SCHORK, A. J., INGASON, A., ASCHARD, H., BRIKELL, I., BULIK, C. M., PETERSEN, L. V., AGERBO, E., GROVE, J. et al. (2023). Multi-PGS enhances polygenic prediction by combining 937 polygenic scores. *Nature communications* **14** 4702.

TRUONG, B., HULL, L. E., RUAN, Y., HUANG, Q. Q., HORNSBY, W., MARTIN, H., VAN HEEL, D. A., WANG, Y., MARTIN, A. R., LEE, S. H. et al. (2024). Integrative polygenic risk score improves the prediction accuracy of complex traits and diseases. *Cell Genomics* **4**.

ZENG, P. and ZHOU, X. (2017). Non-parametric genetic prediction of complex traits with latent Dirichlet process regression models. *Nature communications* **8** 456.
